# Detection of *Mycobacterium tuberculosis* in urine by Xpert MTB/RIF Ultra: A useful adjunctive diagnostic tool in HIV-associated tuberculosis

**DOI:** 10.1016/j.ijid.2018.07.007

**Published:** 2018-10

**Authors:** Rachel R. Atherton, Fiona V. Cresswell, Jayne Ellis, Caleb Skipper, Kiiza K. Tadeo, Gerald Mugumya, Vincent Wadda, David B. Meya, David R. Boulware

**Affiliations:** a Infectious Diseases Institute, Kampala, Uganda; b Clinical Research Department, London School of Hygiene and Tropical Medicine, Keppel Street, London WC1E 7HT, United Kingdom; c Makerere University College of Health Sciences, Upper Mulago Hill Road, Kampala, Uganda; d University of Minnesota, Minneapolis, MN 55455, USA

**Keywords:** *Mycobacterium tuberculosis*, Urine, Xpert MTB/RIF Ultra, Renal tuberculosis

## Abstract

In January 2017, the World Health Organisation recommended the Xpert^®^ MTB/RIF Ultra assay (Ultra) for tuberculosis (TB) diagnosis. Ultra offers improved analytical sensitivity when compared with the initial Xpert^®^ MTB/RIF (Xpert) assay for the detection of *Mycobacterium tuberculosis*. Ultra is therefore likely to be of particular benefit for detecting paucibacillary TB.

We present a case from Uganda demonstrating Ultra positivity in urine from an HIV-infected patient presenting with altered mental status and urinary incontinence, and no other signs of active pulmonary or extrapulmonary TB. This represents the first published instance of a diagnosis of extrapulmonary TB made on the basis of a positive urine Ultra assay.

The use of Ultra on urine may be a useful addition to the diagnostic armamentarium for disseminated TB in persons with HIV co-infection. The diagnostic accuracy of urine Ultra should be characterised further via prospective studies.

## Highlights

• Ultra has detected *Mycobacterium tuberculosis* in the urine of a patient with renal TB.
• Use of Xpert or Ultra on urine is not currently recommended due to lack of evidence.
• Urine Ultra may be useful in the diagnosis of extra-pulmonary TB in persons with HIV.
• Further study is required to characterise the diagnostic accuracy of urine Ultra.

## Introduction

The use of the Xpert^®^ MTB/RIF assay (Xpert) nucleic acid amplification test was first recommended for the diagnosis of tuberculosis (TB) by the World Health Organisation (WHO) in 2010 (World Health Organization, 2014). This fully-automated polymerase chain reaction (PCR) test represented a significant step forward in TB diagnostics. Xpert offered both increased accuracy and speed of diagnosis over the traditional microscopy and culture, as well as the ability to detect rifampicin resistance. In January 2017, Cepheid introduced the next generation of Xpert^®^ named Xpert^®^ MTB/RIF Ultra (Ultra). This offered improved sensitivity over Xpert in the diagnosis of pulmonary TB, most marked in people with HIV co-infection (5.4% higher overall (95% CI +3.3, +8.0%), 13% higher in HIV-infected (95% CI, +6.4, +21%)), although with lower specificity (2.7% lower than Xpert (95% CI −1.7, −3.9)) (Dorman et al., 2018). Sensitivity in TB meningitis was two-fold improved over Xpert (Bahr et al., 2018).

Although introduced for use on sputum samples, and since endorsed by the WHO for use on cerebrospinal fluid (CSF) (World Health Organisation, 2014), the use of Xpert or Ultra with more easily accessible samples (e.g. stool, urine, blood) is not currently recommended due to a lack of evidence (World Health Organization, 2014). Currently, the WHO recommends the use of urine as a diagnostic specimen in TB only with the TB lipoarabinomannan (TB-LAM) antigen lateral flow assay (Alere) (WHO, 2015).

Renal TB is a common form of extra-pulmonary TB in HIV-infected persons and originates primarily from the haematogenous spread of tuberculous bacilli from a respiratory focus. Post-mortem evidence of renal TB microabscesses exists in 50–69% of disseminated TB infections among HIV-infected persons (Lawn and Gupta-Wright, 2016). Urine microscopy and mycobacterial culture remains the gold standard for diagnosis; however, sensitivity is low (with a culture yield reported as around 46% in HIV-negative persons) and results take up to six weeks (Pang et al., 2017). Previous work has shown an increased diagnostic yield in HIV-infected persons (Shafer et al., 1991); however, evidence from the antiretroviral therapy era is limited. A recent study which used Xpert on urine samples demonstrated a 94.6% sensitivity for renal TB when compared to urine mycobacterial culture (Pang et al., 2017).

## Case presentation

A 56-year-old HIV-infected female presented to Kiruddu General Hospital, Kampala, Uganda accompanied by her 17-year-old daughter. Her daughter reported a three-week history of gradual onset confusion, neck pain, and generalised body weakness. In addition, she had urinary incontinence for several months. She reported no systemic symptoms of TB.

She had been on antiretroviral therapy (ART) and prophylactic cotrimoxazole for 12 years. She attended her HIV clinic every 1–2 months, with good compliance. She had undergone two previous changes to her antiretroviral regimen: the first three years prior due to virologic failure, from zidovudine, lamivudine and nevirapine to second line tenofovir, lamivudine and atazanavir/ritonavir. Her second change three months prior to admission was after having discontinued ART for two weeks when developing urinary incontinence. Thereafter, her tenofovir was switched to abacavir. Her most recent HIV viral load was 251 copies/ml with a CD4 of 384 cells/μL (9 months prior to admission). Repeat plasma HIV viral load during the current admission was 1840 copies/mL, and CSF viral load was 32,000 copies/mL.

She was treated for cryptococcal meningitis 12 years prior and had been taking fluconazole secondary prophylaxis since. In addition, she completed treatment for pulmonary TB (confirmed by sputum Xpert) three years prior. She was known to have type II diabetes mellitus, and took regular metformin and glibenclamide.

On admission, she was afebrile with other vital signs within normal range, and a random blood glucose of 7.4 mM (133 mg/dL). On examination, she had a Glasgow coma scale score of 14 (of 15 possible) due to confusion but no focal neurological signs. She had no evidence of wasting and no palpable lymphadenopathy. She had mild generalised abdominal tenderness with no palpable masses or organomegaly, and no abnormalities on thoracic examination.

## Management and outcome

Diagnostic work-up was commenced for altered mental status and possible meningitis; a lumbar puncture was recommended but initially declined by proxy. A serum cryptococcal antigen lateral flow assay (CrAg) was negative so she received three doses of intravenous (IV) ceftriaxone for potential bacterial aetiology, as well as maintenance IV normal saline. A urinary catheter was inserted.

By the fifth day of admission, no improvement had occurred. A lumbar puncture was performed with proxy consent which demonstrated a CSF lymphocytosis of 220 cells/μL, but was otherwise unremarkable (protein 48 mg/dL; glucose error; CrAg negative). CSF microscopy was negative for organisms (including acid-fast bacilli), negative on bacterial culture, and both CSF Xpert and Ultra were negative. CSF mycobacterial growth indicator tube culture was negative after six weeks.

On the sixth day of admission, a catheterised urine sample was negative on TB-LAM assay; however, the centrifuged urine was positive for *M. tuberculosis* by Ultra (‘very low’; Supplemental Table 1). Blood and centrifuged urine were both sent for mycobacterial culture with results negative after six weeks.

On the eighth day of admission, in the absence of any other causative organism or pathology being identified as a cause of her altered mental status and urinary symptoms, the decision was made to commence the patient on anti-tuberculous therapy for treatment of renal TB. A rifabutin-based regimen was started (rifampicin being contraindicated due to pharmacokinetic interaction with atazanavir), and she was discharged on day 14. She was reviewed in clinic on day 20 (at 13 days of TB therapy), and via telephone consultation after 6 weeks, at which point she was noted have improved orientation and mobility and her neck pain had resolved.

## Discussion

We report the first case in which Ultra performed on the urine has been instrumental in identifying disseminated TB in a HIV-infected woman, presenting with non-specific symptoms in a high TB prevalence area. It remains unclear in this instance whether this represents relapse of her prior TB infection or reinfection. We feel it is probable that her disseminated TB infection included the central nervous system in light of her confusion, neck pain and CSF lymphocyte pleocytosis. Against the uniform case definition for TB meningitis she scored 12 points categorising her as a ‘probable’ TBM case (Marais et al., 2010). The negative CSF Xpert and Ultra results demonstrate the imperfect negative predictive value of the assays for CNS TB which can be compounded by collection of small volumes of CSF or infection of the brain tissue or spinal cord without release of TB DNA into the CSF.

The utility of Xpert on urine has already been described (Hillemann et al., 2011, Tortoli et al., 2012), with meta-analysis reporting a summary sensitivity of 0.87 (0.66–0.96) and specificity of 0.91 (0.84–0.95) (Hillemann et al., 2011). A more recent study of concentrated urine in renal TB patients reported sensitivity of 0.95 (0.87–1.00) and specificity of 0.87 (0.81–0.93) when compared to conventional culture (Shafer et al., 1991).

Ultra has an 8-fold lower analytical limit of detection than Xpert (∼15 CFU/mL versus 100–120 CFU/mL respectively) (Chakravortya et al., 2017), so Ultra is potentially more sensitive in paucibacillary TB. So far, Ultra’s improved sensitivity has been demonstrated in the diagnosis of HIV-associated TB meningitis and HIV-associated pulmonary TB (Dorman et al., 2018, Bahr et al., 2018).

Furthermore, specificity of both Xpert and Ultra is increased via a pre-amplification wash step, which ensures that DNA is only detected when associated with tuberculous bacilli (and not trans-renal free DNA or environmental contamination). A positive result in urine is therefore specific for renal TB, likely secondary to disseminated disease. False-positive results are recognised in the sputum of patients previously treated for pulmonary TB, due to persistent viable or non-viable bacilli of Mycobacterium tuberculosis. False positive results on sputum from prior TB infection are often in the ‘trace’ semi-quantitative category (Dorman et al., 2018). The fact that this woman’s result was in the ‘very low’ category adds weight to this being a true positive result, though we cannot exclude this result representing prior renal TB or disseminated tuberculosis.

Urine TB-LAM is currently WHO-recommended for the diagnosis of HIV-associated TB disease in persons with CD4 <200 cells/μL. Those with advanced immunosuppression have higher risk of disease dissemination with consequent renal involvement releasing LAM glycolipid into the urine. A prior study reported good correlation between urine Xpert and TB-LAM, both markers of renal TB (Lawn and Gupta-Wright, 2016, Wood et al., 2012), although more recent data suggest only a small incremental diagnostic yield of urine Xpert over TB-LAM (Gupta-Wright et al., 2018). Whether Ultra will have a greater incremental value deserves exploring. However, in the case reported above, urine TB-LAM was negative, and without a positive Ultra result, the extrapulmonary TB may have been missed. This discordancy in Ultra and TB-LAM results may represent the greater sensitivity of Ultra. Further prospective studies are required to assess whether Ultra is beneficial in conjunction with other TB diagnostics (such as urine TB-LAM) to provide a novel method for diagnosing renal TB and HIV-associated disseminated TB.
