# Supplementary material for: Detection of Mycobacterium tuberculosis in urine by Xpert MTB/RIF Ultra: A useful adjunctive diagnostic tool in HIV-associated tuberculosis
Source: Int J Infect Dis. 2018 Oct;75:92–4. doi: 10.1016/j.ijid.2018.07.007 (PMC6170999; doi:10.1016/j.ijid.2018.07.007)
Supplement: Supplementary file 1 [file mmc1.docx]

**Supplemental Table 1**: Xpert MTB-RIF Ultra assay (v2) results.

| Analyte name | Ct | EndPt | Analyte result | Probe check result |
| --- | --- | --- | --- | --- |
| SPC | 27.8 | 97 | NA | PASS |
| IS1081-IS6110 | 26.2 | 470 | NA | PASS |
| rpoB1 | 38.9 | 50 | POS | PASS |
| rpoB2 | 37.6 | 47 | POS | PASS |
| rpoB3 | 39.3 | 33 | POS | PASS |
| rpoB4 | 0.0 | 12 | NEG | PASS |
